# Dialyzer Classification and Mortality in Hemodialysis Patients: A 3-Year Nationwide Cohort Study

**DOI:** 10.3389/fmed.2021.740461

**Published:** 2021-08-27

**Authors:** Masanori Abe, Ikuto Masakane, Atsushi Wada, Shigeru Nakai, Kosaku Nitta, Hidetomo Nakamoto

**Affiliations:** ^1^The Committee of Renal Data Registry, Japanese Society for Dialysis Therapy, Tokyo, Japan; ^2^Division of Nephrology, Hypertension and Endocrinology, Department of Internal Medicine, Nihon University School of Medicine, Tokyo, Japan; ^3^Department of Nephrology, Yabuki Hospital, Yamagata, Japan; ^4^Department of Nephrology, Kitasaito Hospital, Asahikawa, Japan; ^5^Department of Clinical Engineering, Fujita Health University, Aichi, Japan; ^6^Department of Nephrology, Tokyo Women's Medical University, Tokyo, Japan; ^7^Department of General Internal Medicine, Saitama Medical University, Saitama, Japan

**Keywords:** β_2_-microglobulin clearance, hemodialysis, high-flux dialyzer, low-flux dialyzer, mortality, protein-leaking dialyzer

## Abstract

**Background:** Dialyzers are classified as low-flux, high-flux, and protein-leaking membrane dialyzers internationally and as types I, II, III, IV, and V based on β_2_-microglobulin clearance rate in Japan. Type I dialyzers correspond to low-flux membrane dialyzers, types II and III to high-flux membrane dialyzers, and types IV and V to protein-leaking membrane dialyzers. Here we aimed to clarify the association of dialyzer type with mortality.

**Methods:** This nationwide retrospective cohort study analyzed data from the Japanese Society for Dialysis Therapy Renal Data Registry from 2010 to 2013. We enrolled 238,321 patients on hemodialysis who were divided into low-flux, high-flux, and protein-leaking groups in the international classification and into type I to V groups in the Japanese classification. We assessed the associations of each group with 3-year all-cause mortality using Cox proportional hazards models and performed propensity score matching analysis.

**Results:** By the end of 2013, 55,308 prevalent dialysis patients (23.2%) had died. In the international classification subgroup analysis, the hazard ratio (95% confidence interval) was significantly higher in the low-flux group [1.12 (1.03–1.22), *P* = 0.009] and significantly lower in the protein-leaking group [0.95 (0.92–0.98), *P* = 0.006] compared with the high-flux group after adjustment for all confounders. In the Japanese classification subgroup analysis, the hazard ratios were significantly higher for types I [1.10 (1.02–1.19), *P* = 0.015] and II [1.10 (1.02–1.39), *P* = 0.014] but significantly lower for type V [0.91 (0.88–0.94), *P* < 0.0001] compared with type IV after adjustment for all confounders. These significant findings persisted after propensity score matching under both classifications.

**Conclusions:** Hemodialysis using protein-leaking dialyzers might reduce mortality rates. Furthermore, type V dialyzers are superior to type IV dialyzers in hemodialysis patients.

## Introduction

β_2_-microglobulin (β2MG) is a low-molecular-weight protein (11.8 kDa) that is produced by all cells expressing major histocompatibility class I. It is the main protein component in dialysis-related amyloidosis (1). Because β2MG is exclusively removed by the kidneys, its concentration increases in parallel with declining glomerular filtration rate in chronic kidney disease and shows its highest values in patients on dialysis (2–4). Serum β2MG level is also associated with several comorbid conditions, such as malignancy and inflammation. Higher serum β2MG is associated with higher mortality and rapid decline in kidney function in the general population (5, 6) and non-dialysis-dependent chronic kidney disease patients (7). Regarding hemodialysis patients, *post hoc* analysis of the Hemodialysis (HEMO) study revealed that the serum β2MG level predicted all-cause mortality independently of several confounding factors, including the dialysis prescription and residual kidney function. Indeed, European Best Practice Guidelines recommend the use of β2MG as a marker for middle-molecular-weight uremic toxins and stress its removal in patients on hemodialysis (8). Accordingly, dialyzer types are classified in Japan based on the β2MG clearance rate.

Dialyzers are commonly classified as low- or high-flux membrane dialyzers. Low-flux membrane dialyzers are defined by an ultrafiltration rate <15 mL/mmHg/h and a β2MG clearance rate <15 mL/min (9). They effectively remove small solutes through diffusion, but only negligible amounts of middle-sized solutes, which are considered more toxic and more difficult to remove by diffusion (10). This limitation led to the development of high-flux membrane dialyzers, which are defined by an ultrafiltration rate ≥ 15 mL/mmHg/h and a β2MG clearance rate ≥ 15 mL/min (9). High-flux membranes have high hydraulic permeability and higher solute permeability for middle-sized solutes than low-flux membrane dialyzers. In 2005, to remove an expanded range of larger middle-molecular-weight molecules, protein-leaking membranes with a large pore size were developed in Japan (11). In 2008, more than 90% of Japanese patients on hemodialysis were being treated with this type of dialyzer (10, 12). In Japan, dialyzers are classified into five types based on β2MG clearance: types I, II, III, IV, and V have β2MG clearance rates of <10, ≥10–30, ≥30–50, ≥50–70, and ≥70 mL/min, respectively, at a blood flow rate of 200 mL/min and dialysate flow rate of 500 mL/min (13, 14). In addition, dialyzers are internationally classified into three types: low-flux, high-flux, and protein-leaking.

The aim of this study was to use data from a large-scale registry of dialysis patients in Japan to investigate the impact of dialyzers on clinical outcome in patients undergoing hemodialysis according to the international (i.e., flux-dependent) and Japanese (i.e., β2MG clearance-dependent) classifications.

## Methods

### Data Source

All data analyzed in this study were taken from the Japanese Society for Dialysis Therapy (JSDT) Renal Data Registry (JRDR). These data are collected through a questionnaire-based national survey, the design and methods of which have been reported elsewhere (15, 16). This annual survey has been conducted nationwide in Japan since 1968 and comprises a facility questionnaire completed by staff at dialysis facilities and a patient questionnaire. The survey data have been investigated previously (15, 16), and the JSDT website provides information about the survey's inception, limitations, validity, variables, and questionnaires (17). These national registry data were provided by 4152 of 4226 centers (98.2%) in 2010, 4205 of 4255 centers (98.8%) in 2011, 4233 of 4279 centers (98.9%) in 2012, and 4264 of 4325 centers (98.6%) in 2013; therefore, this registry can be considered representative of Japanese dialysis patients (18, 19).

The data analyzed in this study do not contain personally identifiable information. The study was conducted according to the principles of the Declaration of Helsinki, Japanese privacy protection laws, and the Ethical Guidelines for Medical and Health Research Involving Human Subjects published by the Ministry of Education, Science, and Culture and the Ministry of Health, Labor and Welfare in 2015. The study was approved by the Medicine Ethics Committee of the JSDT. The need for informed consent was waived due to the use of de-identified information. This study is registered with the University Hospital Medical Information Network (UMIN000018641).

### Study Design

The study period for this 3-year retrospective cohort study using JRDR data was December 31, 2010 (18) to December 31, 2013 (19). Data as of December 31, 2010 were defined as baseline data. Eligibility criteria were as follows: age ≥ 18 years; undergoing maintenance dialysis in Japan at the end of 2010, and 3 years of follow-up from 2010 to 2013. Exclusion criteria were dialysis <3 times a week or for <2 h daily, organ transplantation, peritoneal dialysis, and missing data on date of birth, dialysis initiation, type of dialyzer, or outcome. The main outcome measure of this study was time to all-cause mortality during the 3-year observation period. Follow-up ended at death, withdrawal, kidney transplantation, or December 31, 2013, whichever occurred first. Patients were divided into three groups according to the international classification based on flux type—low-flux, high-flux, and protein-leaking—and into five groups according to the Japanese classification based on β2MG clearance—I, II, III, IV, and V—at baseline. The present study thus involved 2 subgroup analyses based on dialyzer criteria in an international classification and in a Japanese classification.

### Definition of Dialyzer Type

In Japan, dialyzer type is defined based on β2MG clearance and divided into five categories—types I to V—according to JSDT guidelines (13). Type I, II, III, IV, and V dialyzers are defined by β2MG clearance rates of <10, ≥10–30, ≥30–50, ≥50–70, and ≥70, respectively. Type I dialyzers are defined as low-flux dialyzers, type II and III dialyzers as high-flux dialyzers, and type IV and V dialyzers as protein-leaking dialyzers according to ultrafiltration rate and β2MG clearance (see Supplementary Table 1 for details of the dialyzer classifications and Supplementary Table 2 for the names and characteristics of the dialyzers used in the present study).

### Covariates and Outcome Data

Baseline patient and laboratory data were collected from the JRDR database in 2010. These variables included age, sex, dialysis duration, modality, body mass index [BMI; calculated as post-hemodialysis body weight (kg)/height (m) squared], cause of end-stage kidney disease, laboratory measures including pre-hemodialysis hemoglobin, serum albumin, phosphate, calcium, intact parathyroid hormone (i-PTH), β2MG, and C-reactive protein (CRP) levels, and history of myocardial infarction, cerebral hemorrhage, cerebral infarction, and limb amputation. Shinzato's formula was used to calculate single-pooled Kt/V and normalized protein catabolic rate (nPCR) (20, 21).

### Statistical Methods

Data are summarized as proportions with the mean ± standard deviation or median [interquartile range] as appropriate. Categorical variables were analyzed using the chi-square test, and continuous variables were compared using Student's *t*-test. Categorical data were compared between groups using repeated-measures ANOVA and Tukey's honestly significant difference test or the Kruskal–Wallis test, as appropriate.

Survival according to dialyzer type was estimated using the Kaplan–Meier method and compared using the log-rank test. To examine whether baseline basic factors, including age, sex, cause of end-stage kidney disease, dialysis duration, and comorbid cardiovascular disease (CVD), predicted survival for up to 3 years of follow-up, we performed survival analyses with Cox proportional hazards regression. Additional analyses were carried out after adjusting for dialysis-related factors assessed by Kt/V and β2MG. Analyses were additionally performed with adjustment for nutrition- and inflammation-related factors, including BMI, nPCR, percent creatinine generation rate (%CGR), and serum albumin, hemoglobin, phosphate, calcium, i-PTH, and CRP levels. In the analyses, age, β2MG, CRP levels, and hemoglobin levels were treated as continuous variables. In the final analysis, associations were examined between all-cause mortality and the three flux types and the five dialyzer types according to the β2MG clearance. Patients were divided into three dialyzer groups in the international classification subgroup analysis and into five groups in the Japanese classification subgroup analysis. These analyses were performed with adjustment for the abovementioned basic factors, as well as dialysis-related factors and nutrition- and inflammation-related factors measured at baseline. The reference group was the high-flux dialyzer group in the international classification subgroup analysis because this type of dialyzer is recommended in National Kidney Foundation Kidney Disease Outcomes Quality Initiative (KDOQI) and European Best Practice guidelines (22, 23) and the type IV dialyzer group in the Japanese classification subgroup analysis because it is the most widely used dialyzer in Japan.

In addition, propensity score matching was used to adjust for significant baseline covariates. The abovementioned basic factors, dialysis-related factors, and nutrition- and inflammation-related factors were used to calculate propensity scores, which were then used in univariate Cox proportional hazards regression analysis. Patients with a high-flux dialyzer and type IV dialyzer (reference group) were matched in a 1:1 ratio with the other types of dialyzers in each classification. Propensity scores were derived from age, sex, dialysis vintage, comorbid CVD and diabetes, BMI, Kt/V, β2MG, nPCR, %CGR, and serum albumin, hemoglobin, phosphate, calcium, i-PTH, and CRP levels. All-cause mortality was also compared in propensity score-matched patients.

When appropriate, missing covariate data were imputed by a conventional method for multivariate regression. All analyses were performed using JMP® version 13.0 (SAS Institute, Cary, NC). The level of significance was set as *P* < 0.05.

## Results

**Figure 1:**
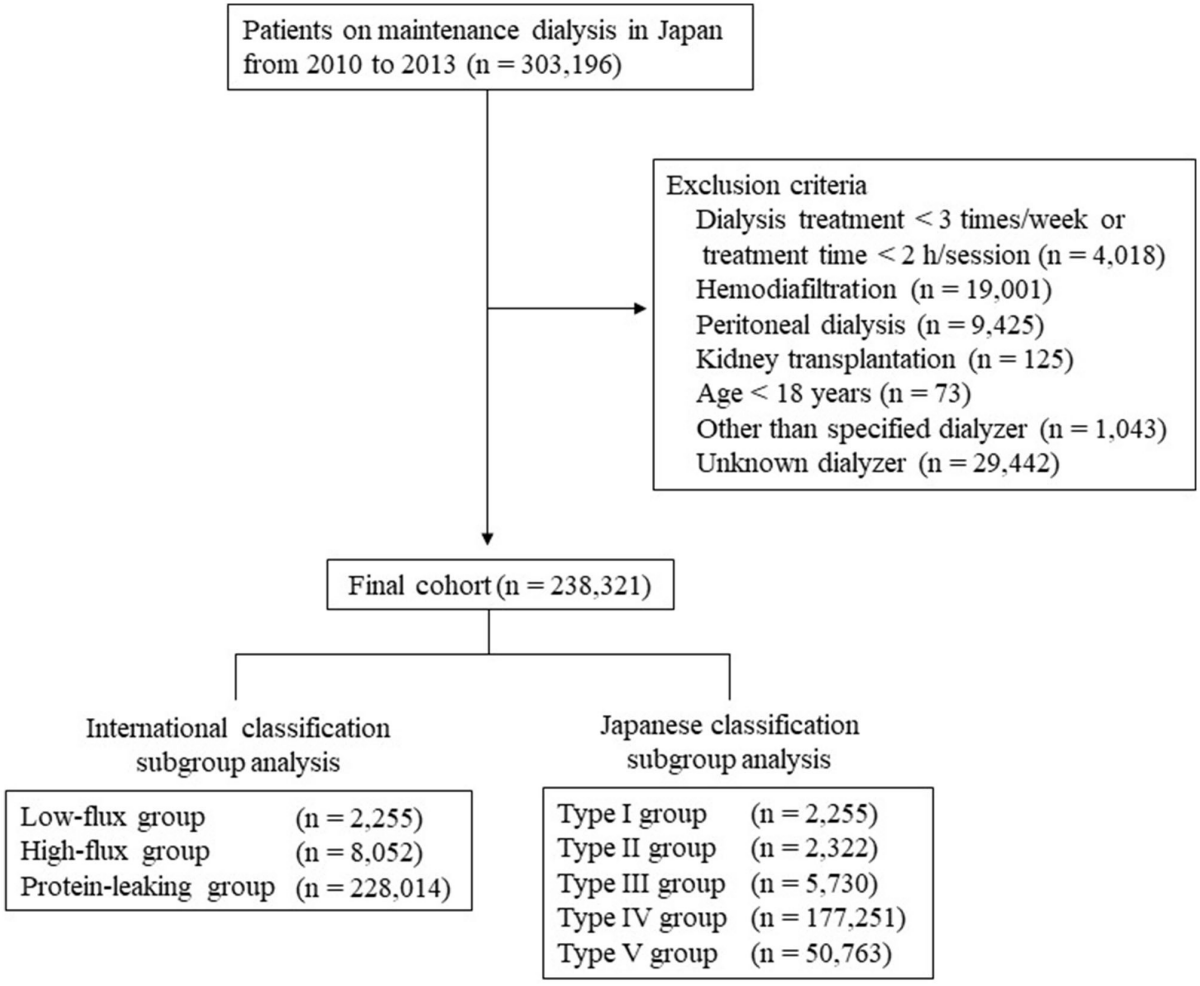
Flowchart of study participants.

Figure 1 summarizes the data extraction process. The original data set included 303,196 patients at the end of 2010, and 238,321 patients remained after exclusions. Table 1 shows the baseline characteristics of the 238,321 patients (age, 66.3 ± 12.4 years; male, 62.7%; median dialysis duration, 6 years) with data on dialyzer type. The underlying conditions comprised chronic glomerulonephritis in 38.6%, diabetic nephropathy in 36.2%, nephrosclerosis in 8.4%, polycystic kidney disease in 3.4%, and other or unknown in 13.4%. Subgroup analyses were performed according to the international and Japanese classifications. Supplementary Table 3 shows the proportions of categorical variables. Mean follow-up duration was 2.6 ± 0.8 years. During the observation period, 55,308 deaths (23.2%) were recorded: 23,720 cardiovascular-related deaths, 10,902 infection-related deaths, 5,325 cancer-related deaths, and 15,361 other deaths.

**Table 1:** Demographic, clinical, and laboratory values at baseline for the 238,321 hemodialysis patients included in this study.

**Variables**	**Values**
Number of patients (% female)	238,321 (37.3)
Age (years)	66.3 ± 12.4
Dialysis duration (years)	6 [3–11]
Primary kidney disease (%)	
Glomerulonephritis	38.6
Diabetic nephropathy	36.2
Nephrosclerosis	8.4
Polycystic kidney disease	3.4
Others	13.4
Comorbid CVD (%)	26.5
Coronary artery disease	8.1
Ischemic stroke	15.6
Hemorrhagic stroke	5.2
Limb amputation	3.1
Body mass index (kg/m^2^)	21.3 ± 3.7
Hemoglobin (g/dL)	10.5 ± 1.6
Calcium (mg/dL)	8.9 ± 0.8
Phosphate (mg/dL)	5.2 ± 1.5
Intact PTH (pg/mL)	116 [59–198]
C-reactive protein (mg/dL)	0.1 [0.1–0.4]
β_2_-microglobulin (mg/L)	26.7 ± 7.0
Albumin (g/dL)	3.7 ± 0.4
Kt/V	1.43 ± 0.28
nPCR (g/kg/day)	0.87 ± 0.18
%CGR (%)	93.5 ± 28.5

*%CGR, percent creatinine generation rate; CVD, cardiovascular disease; nPCR, normalized protein catabolic rate; PTH, parathyroid hormone*.

### Predictors of All-Cause Mortality in 238,321 Hemodialysis Patients

The hazard ratios (HRs) and 95% confidence intervals for variables that were evaluated as potential predictors of mortality in hemodialysis patients are shown in Supplementary Table 4. Of basic factors, significant predictors of mortality were male sex, older age, longer dialysis vintage, comorbid CVD, and presence of diabetes mellitus (DM). Regarding dialysis-related factors, lower mortality risk was associated with higher single-pool Kt/V and lower β2MG levels. Furthermore, for nutrition- and inflammation-related factors, higher mortality was associated with poor nutritional status, indicated by lower hemoglobin, serum albumin, BMI, nPCR, and %CGR values, and with increased inflammatory status, indicated by higher CRP levels.

### Clinical and Demographic Characteristics of the Three Dialyzer Groups in the International Classification

Table 2 shows the patient demographics and characteristics in each dialyzer group in the international classification subgroup analysis: most patients received hemodialysis with protein-leaking dialyzers (95.6%), followed by high-flux dialyzers (3.4%) and low-flux dialyzers (1.0%). Patients treated using low-flux dialyzers were older and more likely to be female and had higher rates of comorbid CVD and DM and lower BMI. In contrast, patients treated using protein-leaking dialyzers were younger and more likely to be male and had lower rates of comorbid CVD and DM and higher Kt/V, nPCR, and %CGR.

**Table 2:** Demographic, clinical, and laboratory values in 238,321 hemodialysis patients according to dialyzer type in the international classification.

	**Low-flux**	**High-flux**	**Protein-leaking**	***P* value**
*n* (%)	2,255 (1.0)	8,052 (3.4)	228,014 (95.6)	
Age (years)	74.8 ± 10.8	70.6 ± 12.3	66.2 ± 12.4	<0.0001
Sex (% female)	53.3	43.8	37.2	<0.0001
Dialysis duration (years)	3 [1–6]	4 [2–9]	6 [3–11]	<0.0001
Presence of DM (%)	50.9	46.8	43.4	<0.0001
Comorbid CVD (%)	35.1	28.7	26.6	<0.0001
Coronary artery disease	10.6	9.2	8.2	
Ischemic stroke	23.1	17.2	15.5	
Hemorrhagic stroke	6.3	5.5	5.2	
Limb amputation	3.6	3.6	3.1	
BMI (kg/m^2^)	19.9 ± 3.6	20.7 ± 3.6	21.3 ± 3.6	<0.0001
Hemoglobin (g/dL)	10.0 ± 1.4	10.2 ± 1.3	10.5 ± 1.3	<0.0001
Serum albumin (g/dL)	3.4 ± 0.5	3.5 ± 0.5	3.7 ± 0.4	<0.0001
Calcium (mg/dL)	8.6 ± 0.9	8.8 ± 0.8	8.9 ± 0.8	<0.0001
Phosphate (mg/dL)	4.9 ± 1.6	5.1 ± 1.5	5.2 ± 1.4	<0.0001
Intact PTH (pg/mL)	114 [55–197]	115 [58–202]	116 [59–197]	<0.0001
β_2_-microglobulin (mg/L)	29.0 ± 11.3	27.8 ± 8.4	26.6 ± 7.0	<0.0001
C-reactive protein (mg/dL)	0.2 [0.1–0.8]	0.1 [0.1–0.6]	0.1 [0.1–0.4]	<0.0001
Kt/V	1.27 ± 0.29	1.37 ± 0.29	1.44 ± 0.28	<0.0001
nPCR (g/kg/day)	0.79 ± 0.20	0.83 ± 0.18	0.87 ± 0.18	<0.0001
%CGR (%)	71.6 ± 29.8	83.3 ± 30.7	93.7 ± 28.4	<0.0001
Mortality rate (/person-years)	0.21	0.11	0.09	<0.0001

*%CGR, percent creatinine generation rate; CVD, cardiovascular disease; DM, diabetes mellitus; nPCR, normalized protein catabolic rate; PTH, parathyroid hormone*.

### Associations of the Three Flux Dialyzer Groups With All-Cause Mortality

**Figure 2:**
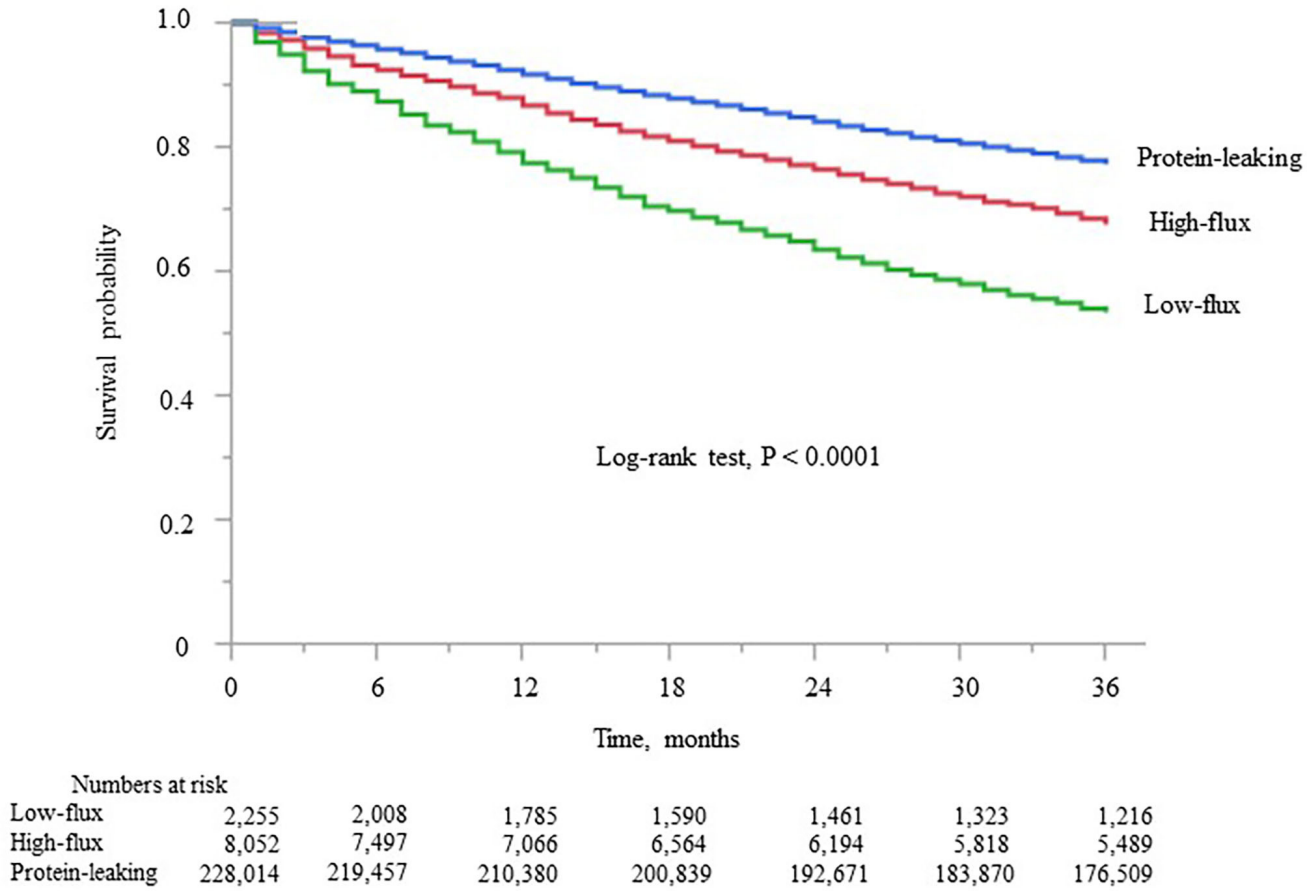
Kaplan–Meier survival curve for all-cause mortality in the three dialyzer type groups in the international classification.

Kaplan–Meier analysis showed that survival steadily deteriorated as the dialyzer type increased (log-rank test, *P* < 0.0001; Figure 2). Compared with the high-flux dialyzer group (reference), the low-flux dialyzer group had a higher unadjusted HR (95% confidence interval) for all-cause mortality of 1.88 (1.76–2.00). In contrast, the protein-leaking dialyzer group showed a lower unadjusted HR for all-cause mortality of 0.78 (0.74–0.82) (Supplementary Table 5).

**Figure 3:**
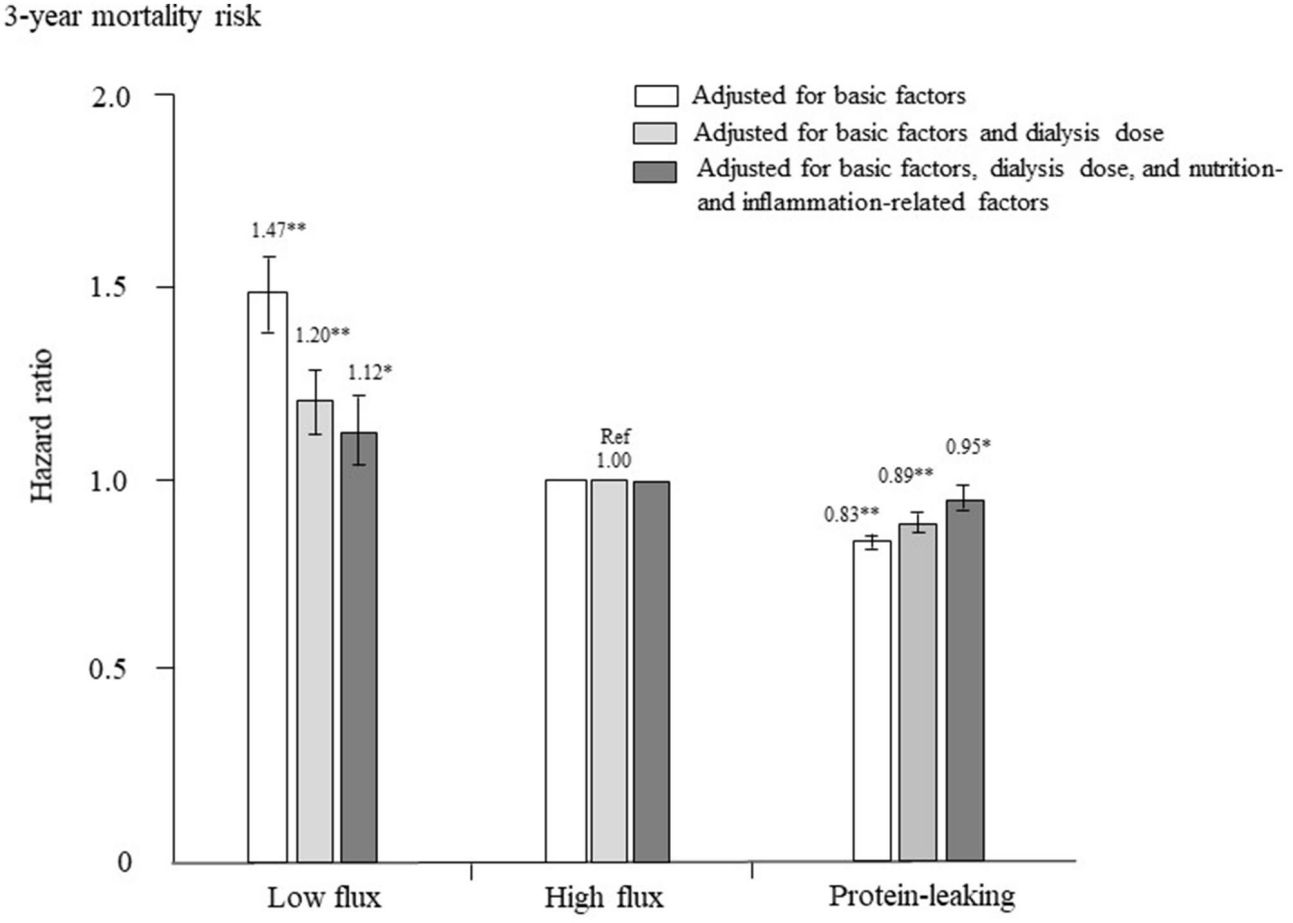
Hazard ratios for all-cause mortality among the three dialyzer types in 238,321 patients undergoing hemodialysis, determined using standard Cox proportional hazards regression. White bars are adjusted for basic factors, including age, sex, dialysis vintage, presence/absence of diabetes mellitus, and presence/absence of cardiovascular complications. Gray bars are adjusted for dialysis dose, as assessed by Kt/V and β_2_-microglobulin levels, in addition to basic factors. Dark gray bars are adjusted for basic factors, dialysis dose, and nutrition- and inflammation-related factors, including body mass index, levels of C-reactive protein, hemoglobin, calcium, phosphate, intact parathyroid hormone, and serum albumin, normalized protein catabolic rate, and percent creatinine generation rate. *P < 0.01, **P < 0.0001 vs. the high-flux dialyzer group (reference). Error bars correspond to 95% confidence intervals.

The adjusted HRs for all-cause mortality in each group are shown in Figure 3. After adjustment for basic factors, including age, sex, dialysis duration, history of CVD, and presence or absence of DM, the HRs of the low-flux and protein-leaking groups, compared with the high-flux group (reference), were 1.47 (1.37–1.57) and 0.83 (0.81–0.85), respectively. After adjustment for basic factors and dialysis-related factors, including Kt/V and β2MG, the HRs of the low-flux and protein-leaking groups, compared with the high-flux group, were 1.20 (1.31–1.50) and 0.89 (0.86–0.91), respectively. Finally, after adjustment for basic factors, dialysis-related factors, and nutrition- and inflammation-related factors, including BMI, hemoglobin, nPCR, %CGR, and serum albumin and CRP levels, the low-flux group had a significantly higher HR of 1.12 (1.03–1.22, *P* = 0.009), whereas the protein-leaking group had a lower HR of 0.95 (0.92–0.98, *P* = 0.006).

### Clinical and Demographic Characteristics of the Five Dialyzer Groups in the Japanese Classification

Table 3 shows the patient demographics and characteristics in each dialyzer group: most patients received hemodialysis with type IV dialyzers (74.3%), followed by type V (21.3%), type III (2.4%), type II (1.0%), and type I (1.0%). Patients treated using type I dialyzers were older and more likely to be female and had higher rates of comorbid CVD and DM and lower BMI. In contrast, patients treated using type V dialyzers were younger and more likely to be male and had lower rates of comorbid CVD and DM and higher Kt/V, nPCR, and %CGR.

**Table 3:** Demographic, clinical, and laboratory values in 238,321 hemodialysis patients according to dialyzer type in the Japanese classification.

	**I**	**II**	**III**	**IV**	**V**	***P* value**
*n* (%)	2,255 (1.0)	2,322 (1.0)	5,730 (2.4)	177,251 (74.3)	50,763 (21.3)	
Age (years)	74.8 ± 10.8	74.2 ± 11.4	69.2 ± 12.4	67.2 ± 12.3	62.6 ± 12.4	<0.0001
Sex (% female)	53.3	49.6	41.4	39.0	31.0	<0.0001
Dialysis duration (years)	3 [1–6]	3 [1–7]	5 [2–9]	6 [3–10]	7 [4–13]	<0.0001
Presence of DM (%)	50.9	47.8	46.2	44.5	39.4	<0.0001
Comorbid CVD (%)	35.1	32.3	27.4	27.6	23.0	<0.0001
Coronary artery disease	10.6	9.6	8.9	8.4	7.3	
Ischemic stroke	23.1	19.8	16.3	16.3	12.8	
Hemorrhagic stroke	6.3	7.0	5.0	5.4	4.5	
Limb amputation	3.6	3.9	3.4	3.2	2.9	
BMI (kg/m^2^)	19.9 ± 3.6	20.1 ± 3.5	20.9 ± 3.5	21.2 ± 3.6	21.8 ± 3.8	<0.0001
Hemoglobin (g/dL)	10.0 ± 1.4	10.1 ± 1.4	10.3 ± 1.3	10.5 ± 1.3	10.6 ± 1.2	<0.0001
Serum albumin (g/dL)	3.4 ± 0.5	3.4 ± 0.5	3.6 ± 0.4	3.7 ± 0.4	3.7 ± 0.4	<0.0001
Calcium (mg/dL)	8.6 ± 0.9	8.7 ± 0.9	8.8 ± 0.8	8.9 ± 0.8	9.0 ± 0.8	<0.0001
Phosphate (mg/dL)	4.9 ± 1.6	4.9 ± 1.5	5.2 ± 1.5	5.2 ± 1.4	5.4 ± 1.5	<0.0001
Intact PTH (pg/mL)	114 [55–197]	108 [57–191]	119 [59–206]	114 [57–195]	122 [64–204]	<0.0001
β_2_-microglobulin, (mg/L)	29.0 ± 11.3	28.2 ± 10.1	27.7 ± 7.7	26.6 ± 7.0	26.9 ± 6.8	<0.0001
C-reactive protein (mg/dL)	0.2 [0.1–0.8]	0.2 [0.1–0.7]	0.1 [0.1–0.5]	0.1 [0.1–0.4]	0.1 [0.1–0.3]	<0.0001
Kt/V	1.27 ± 0.29	1.30 ± 0.29	1.40 ± 0.28	1.43 ± 0.28	1.45 ± 0.28	<0.0001
nPCR (g/kg/day)	0.79 ± 0.20	0.79 ± 0.18	0.84 ± 0.19	0.86 ± 0.18	0.89 ± 0.18	<0.0001
%CGR (%)	71.6 ± 29.8	72.4 ± 29.7	87.3 ± 30.0	91.9 ± 28.8	99.8 ± 26.2	<0.0001
Mortality rate (/person-years)	0.22	0.20	0.11	0.09	0.06	<0.0001

*%CGR, percent creatinine generation rate; CVD, cardiovascular disease; DM, diabetes mellitus; nPCR, normalized protein catabolic rate; PTH, parathyroid hormone*.

### Associations of the Five Dialyzer Groups With All-Cause Mortality in the Japanese Classification

**Figure 4:**
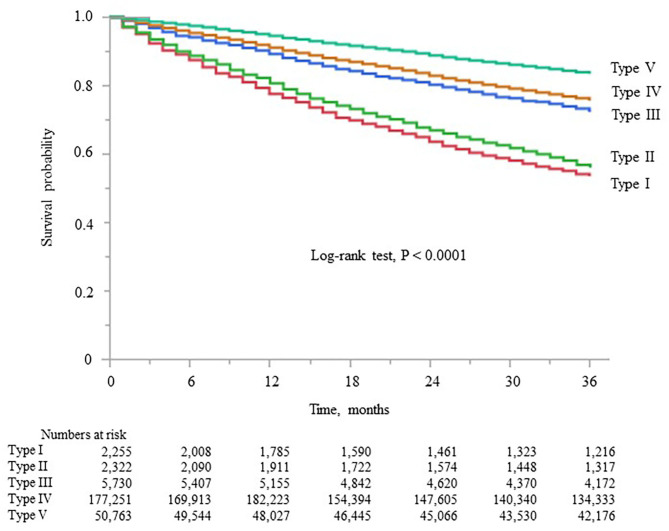
Kaplan–Meier survival curve for all-cause mortality in the five dialyzer type groups in the Japanese classification.

Kaplan–Meier analysis showed that survival steadily deteriorated as dialyzer type increased (log-rank test, *P* < 0.0001; Figure 4), except for type V. Compared with the type IV group (reference), the type I, II, and III groups showed unadjusted HRs for all-cause mortality of 2.35 (2.21–2.49), 2.09 (1.95–2.21), and 1.17 (1.11–1.23), respectively (Supplementary Table 6). The type V group had a significantly lower HR of 0.64 (0.63–0.65). During the 627,538 person-years of follow-up, the mortality rate was significantly and consistently lower in the groups with dialyzers providing higher β2MG clearance (Table 3).

**Figure 5:**
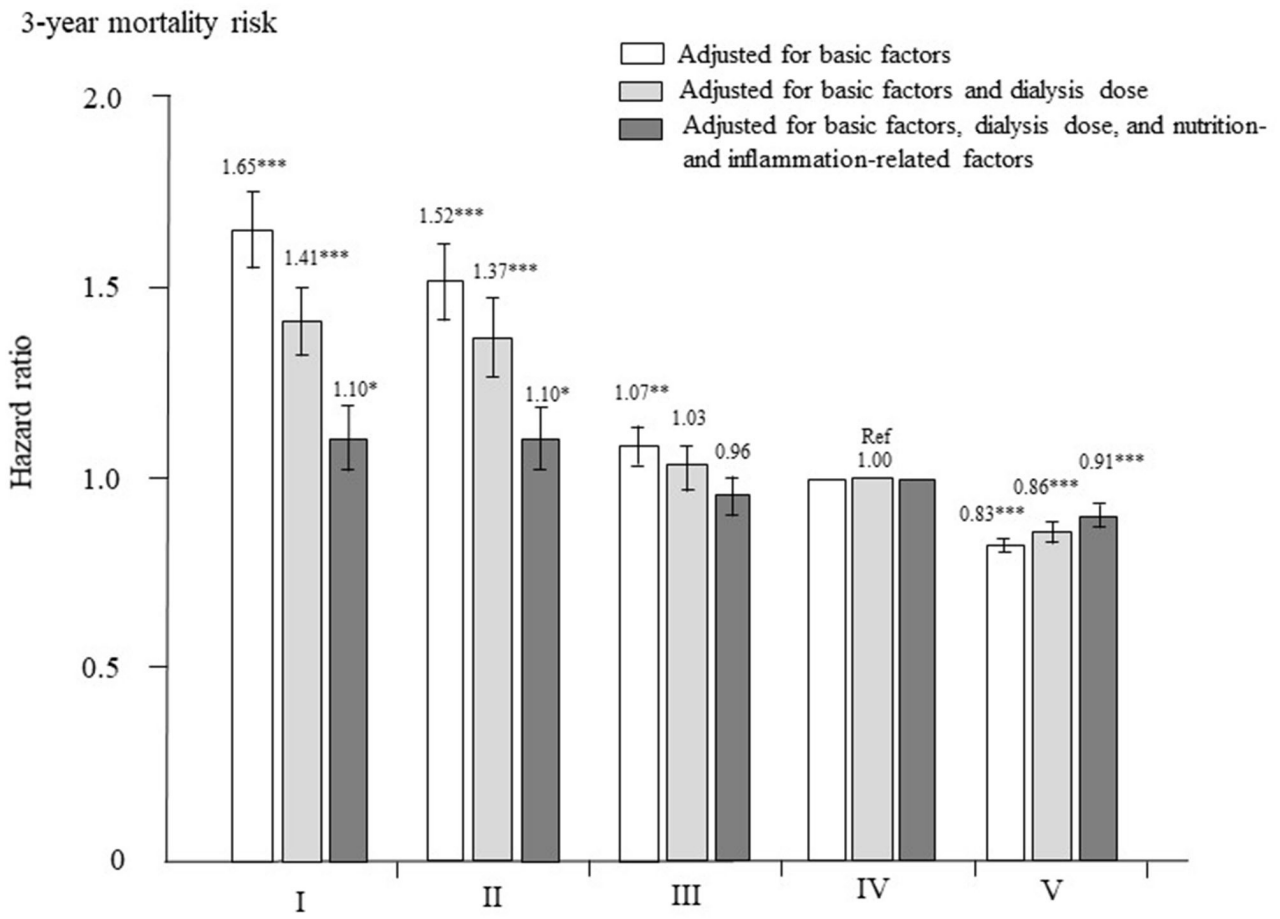
Hazard ratios for all-cause mortality among the five dialyzer types in 238,321 patients undergoing hemodialysis, determined using standard Cox proportional hazards regression. White bars are adjusted for basic factors, including age, sex, dialysis vintage, presence/absence of diabetes mellitus, and presence/absence of cardiovascular complications. Gray bars are adjusted for dialysis dose, as assessed by Kt/V and β_2_-microglobulin levels, in addition to basic factors. Dark gray bars are adjusted for basic factors, dialysis dose, and nutrition- and inflammation-related factors, including body mass index, levels of C-reactive protein, hemoglobin, calcium, phosphate, intact parathyroid hormone, and serum albumin, normalized protein catabolic rate, and percent creatinine generation rate. *P < 0.05, **P < 0.01, ***P < 0.0001 vs. the type IV dialyzer group (reference). Error bars correspond to 95% confidence intervals.

The adjusted HRs for all-cause mortality in each group are shown in Figure 5. After adjustment for basic factors, the HRs of the type I, II, and III groups, compared with the type IV group (reference), were 1.65 (1.55–1.75), 1.52 (1.42–1.62), and 1.07 (1.02–1.12), respectively. The type V group had a significantly lower HR of 0.83 (0.81–0.85). After adjustment for basic factors and dialysis-related factors, the HRs of the type I, II, and III groups, compared with the type IV group, were 1.41 (1.31–1.50), 1.37 (1.27–1.47), and 1.03 (0.97–1.09), respectively. The type III group showed no significant difference from the type IV group, whereas the type V group had a significantly lower HR of 0.86 (0.83–0.88). Finally, after adjustment for basic factors, dialysis-related factors, and nutrition- and inflammation-related factors, the HR of the type III group did not differ significantly from that of the type IV group, but the type I and II groups had significantly higher HRs of 1.10 (1.02–1.19, *P* = 0.015) and 1.10 (1.02–1.39, *P* = 0.014), respectively, whereas the type V group had a lower HR of 0.91 (0.88–0.94, *P* < 0.0001).

### Propensity Score Matching Analysis

**Figure 6:**
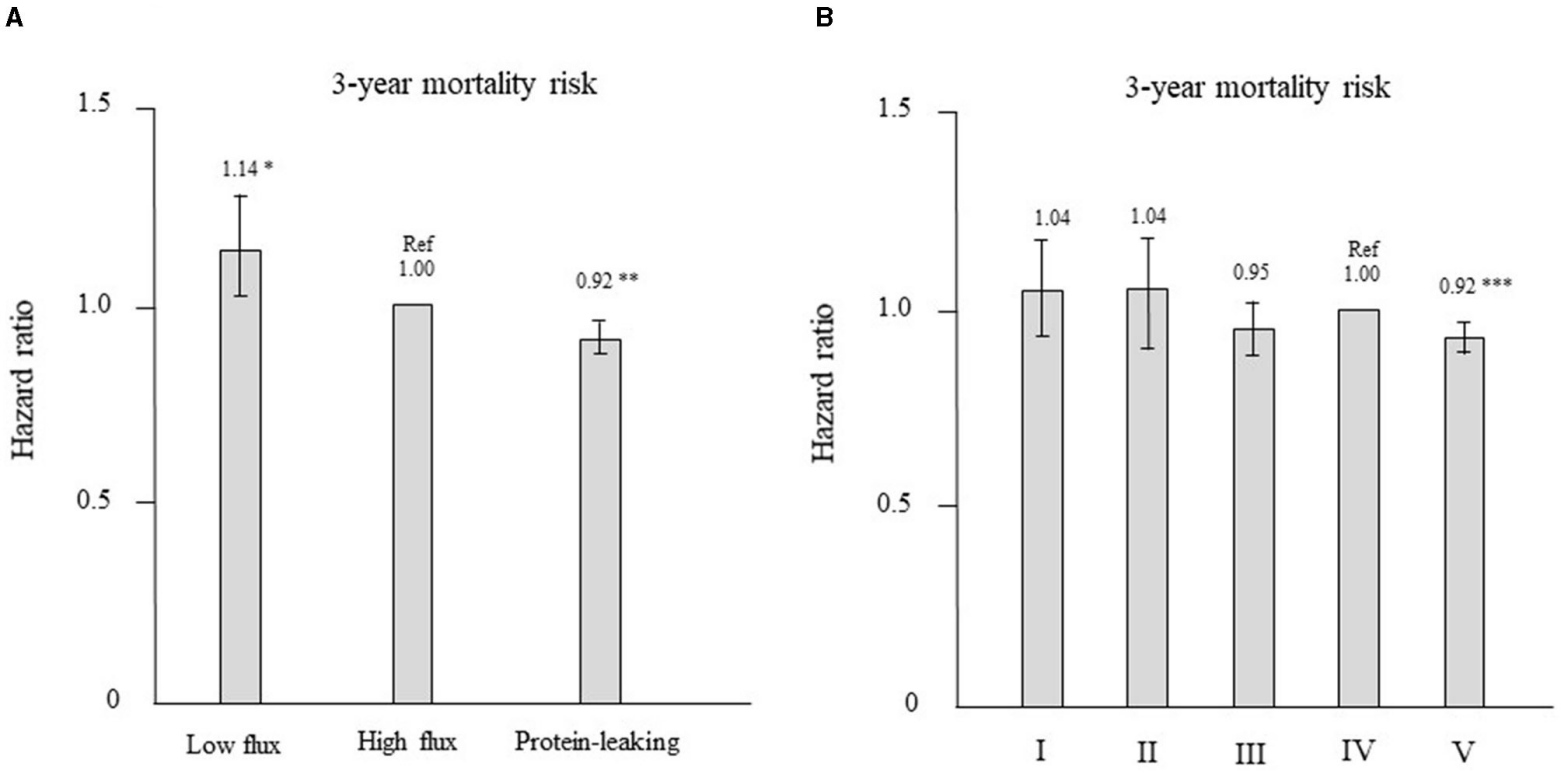
**(A)** Hazard ratios for all-cause mortality after propensity score matching for the 3 types of dialyzers in the international classification (reference: high-flux dialyzer), determined using Cox proportional hazards regression. *P < 0.05, **P < 0.01 vs. the high-flux dialyzer. Error bars correspond to 95% confidence intervals. **(B)** Hazard ratios of all-cause mortality after propensity score matching for the 5 types of dialyzers in the Japanese classification (reference: type IV dialyzer), determined using Cox proportional hazards regression. ***P < 0.001 vs. the type IV dialyzer. Error bars correspond to 95% confidence intervals.

Patients who were treated with high-flux dialyzers were matched with those treated with other types of dialyzers in a 1:1 ratio according to propensity scores. After propensity score matching, 1,229 and 6,214 patient pairs were matched in the low-flux and protein-leaking dialyzer groups, respectively. Table 4 shows the patient characteristics and clinical data at baseline in the high-flux group and in each corresponding group after propensity score matching. There were no significant differences in any variables. As shown in Figure 6A, compared with the high-flux group, the low-flux group had a higher HR [1.14 (1.02–1.26), *P* = 0.022]. However, the protein-leaking group had a significantly lower HR [0.92 (0.87–0.97), *P* = 0.006].

**Table 4:** Baseline characteristics after propensity score matching between high-flux dialyzers (reference) and other dialyzer types.

	**Matched**			**Matched**		
	**Low-flux**	**High-flux**	***P* value**	**Protein-leaking**	**High-flux**	***P* value**
*n* (%)	1,229	1,229	-	6,214	6,214	-
Age (years)	75.2 ± 10.6	75.3 ± 10.0	0.730	70.7 ± 11.5	70.7 ± 12.1	0.815
Sex (% female)	49.5	50.7	0.518	42.9	43.6	0.322
Dialysis duration (years)	3 [1–7]	4 [2–7]	0.934	5 [2–9]	5 [2–9]	0.938
Presence of DM (%)	40.7	38.5	0.513	26.3	26.2	0.869
Comorbid CVD (%)	36.8	35.2	0.401	29.4	29.2	0.828
Body mass index (kg/m^2^)	19.9 ± 3.8	19.9 ± 3.6	0.957	20.7 ± 3.5	20.7 ± 3.5	0.570
Hemoglobin (g/dL)	9.9 ± 1.5	9.9 ± 1.4	0.413	10.2 ± 1.4	10.1 ± 1.4	0.720
Serum albumin (g/dL)	3.3 ± 0.5	3.3 ± 0.5	0.775	3.5 ± 0.5	3.5 ± 0.5	0.721
Calcium (mg/dL)	8.6 ± 0.9	8.6 ± 0.9	0.597	8.8 ± 0.8	8.8 ± 0.8	0.748
Phosphate (mg/dL)	4.9 ± 1.7	4.8 ± 1.6	0.569	5.1 ± 1.5	5.1 ± 1.5	0.521
Intact PTH (pg/mL)	110 [54–189]	97 [53–178]	0.861	107 [53–188]	107 [54–192]	0.179
β_2_-microglobulin (mg/L)	30.0 ± 11.8	29.8 ± 9.7	0.647	29.0 ± 8.3	29.2 ± 8.6	0.349
C-reactive protein (mg/dL)	1.0 [0.5–2.6]	1.0 [0.5–2.5]	0.421	0.1 [0.1–0.6]	0.1 [0.1–0.6]	0.386
Kt/V	1.24 ± 0.30	1.26 ± 0.29	0.213	1.35 ± 0.28	1.35 ± 0.29	0.979
nPCR (g/kg/day)	0.78 ± 0.20	0.78 ± 0.19	0.337	0.83 ± 0.18	0.83 ± 0.18	0.388
%CGR (%)	66.4 ± 30.0	68.9 ± 31.2	0.215	83.4 ± 29.7	83.2 ± 30.3	0.826

*%CGR, percent creatinine generation rate; CVD, cardiovascular disease; DM, diabetes mellitus; nPCR, normalized protein catabolic rate; PTH, parathyroid hormone*.

Patients who were treated with type IV dialyzers were matched with those treated with other types of dialyzers in a 1:1 ratio according to propensity scores. After propensity score matching, 1,214, 1,075, 3,029, and 30,832 patient pairs were matched in the type I, II, III, and V dialyzer groups, respectively. Table 5 shows the patient characteristics and clinical data at baseline in the type IV group and in each corresponding group after propensity score matching. No significant differences were noted in any variables. As shown in Figure 6B, compared with the type IV group, the type I, II, and III groups showed no significant differences in HR. However, the type V group had a significantly lower HR [0.92 (0.89–0.95), *P* = 0.0001].

**Table 5:** Baseline characteristics after propensity score matching between type IV dialyzers (reference) and other dialyzer types.

	**Matched**			**Matched**			**Matched**			**Matched**		
	**I**	**IV**	***P* value**	**II**	**IV**	***P* value**	**III**	**IV**	***P* value**	**V**	**IV**	***P* value**
*n* (%)	1,214	1,214	-	1,075	10,75	-	3,029	3,029	-	30,832	30,832	-
Age (years)	74.9 ± 10.9	75.2 ± 10.2	0.667	74.0 ± 11.7	74.4 ± 10.5	0.500	69.2 ± 12.2	69.2 ± 11.8	0.956	62.5 ± 12.6	62.4 ± 12.4	0.757
Sex (% female)	43.6	45.1	0.438	49.0	50.1	0.605	41.4	40.9	0.676	30.9	30.9	0.882
Dialysis duration (years)	3 [1–7]	4 [2–7]	0.676	3 [2–7]	4 [2–8]	0.694	5 [2–9]	5 [3–9]	0.753	7 [3–13]	7 [4–13]	0.146
Presence of DM (%)	49.6	50.4	0.685	49.5	50.1	0.763	45.6	45.3	0.816	39.6	39.5	0.699
History of CVD (%)	34.8	36.2	0.445	32.3	33.4	0.582	29.4	28.8	0.591	23.3	23.0	0.375
BMI (kg/m^2^)	19.8 ± 3.7	19.8 ± 3.6	0.831	20.1 ± 3.4	20.0 ± 3.3	0.371	20.9 ± 3.5	20.9 ± 3.5	0.977	21.9 ± 3.9	21.9 ± 3.7	0.891
Hemoglobin (g/dL)	10.1 ± 1.4	10.1 ± 1.4	0.721	10.2 ± 1.2	10.2 ± 1.2	0.912	10.4 ± 1.2	10.4 ± 1.3	0.825	10.7 ± 1.2	10.7 ± 1.2	0.668
Serum albumin (g/dL)	3.4 ± 0.5	3.5 ± 0.5	0.864	3.4 ± 0.5	3.4 ± 0.5	0.774	3.6 ± 0.4	3.7 ± 0.4	0.924	3.7 ± 0.4	3.7 ± 0.4	0.241
Calcium (mg/dL)	8.7 ± 0.8	8.7 ± 0.8	0.763	8.7 ± 0.8	8.7 ± 0.8	0.357	8.9 ± 0.8	8.9 ± 0.8	0.356	9.0 ± 0.8	9.0 ± 0.8	0.135
Phosphate (mg/dL)	4.9 ± 1.5	4.8 ± 1.5	0.239	5.0 ± 1.4	5.0 ± 1.4	0.685	5.2 ± 1.5	5.2 ± 1.5	0.107	5.4 ± 1.4	5.4 ± 1.4	0.616
Intact PTH (pg/mL)	109 [54–191]	101 [50–172]	0.879	110 [54–176]	101 [50–171]	0.227	119 [59–202]	112 [57–188]	0.810	125 [64–206]	122 [62–207]	0.619
β2MG (mg/L)	29.2 ± 11.2	28.6 ± 10.9	0.187	27.6 ± 10.1	27.9 ± 9.4	0.479	28.0 ± 7.7	28.0 ± 7.5	0.905	27.0 ± 6.9	27.0 ± 6.8	0.298
CRP (mg/dL)	0.2 [0.1–0.7]	0.2 [0.1–0.9]	0.241	0.1 [0.1–0.6]	0.2 [0.1–0.7]	0.699	0.1 [0.1–0.4]	0.1 [0.1–0.5]	0.946	0.1 [0.1–0.3]	0.1 [0.1–0.3]	0.583
Kt/V	1.30 ± 0.28	1.30 ± 0.29	0.911	1.31 ± 0.28	1.31 ± 0.28	0.541	1.40 ± 0.27	1.40 ± 0.28	0.911	1.45 ± 0.30	1.44 ± 0.29	0.119
nPCR (g/kg/day)	0.80 ± 0.20	0.79 ± 0.19	0.339	0.78 ± 0.17	0.79 ± 0.17	0.231	0.85 ± 0.18	0.85 ± 0.18	0.867	0.90 ± 0.17	0.90 ± 0.17	0.311
%CGR (%)	71.6 ± 29.9	69.8 ± 28.4	0.100	72.8 ± 30.2	73.8 ± 28.3	0.414	90.5 ± 29.1	91.6 ± 28.5	0.117	105.2 ± 22.8	105.5 ± 23.1	0.596

*%CGR, percent creatinine generation rate; β2MG, β2-microglobulin; BMI, body mass index; CRP, C-reactive protein; CVD, cardiovascular disease; DM, diabetes mellitus; Hb, hemoglobin; nPCR, normalized protein catabolic rate; PTH, parathyroid hormone*.

## Discussion

This observational study, which was conducted using a large-scale registry of 238,321 Japanese hemodialysis patients during a 3-year follow-up, revealed that treatment with protein-leaking dialyzers was significantly associated with lower all-cause mortality. Mortality was compared among the three types of flux dialyzers with adjustment for predictive factors. After full adjustment for predictive factors and propensity score matching, the HR was significantly lower in the protein-leaking group than in the high-flux dialyzer group (reference). Furthermore, this study revealed the superiority of type V dialyzers over type IV dialyzers, even though both are categorized in the same protein-leaking category. A major strength of the present study is its large sample size and use of all current types of dialyzers. This study is the first to suggest that mortality risk might be improved in hemodialysis patients by using protein-leaking dialyzers, particularly type V dialyzers, which are defined as those with a β2MG clearance of ≥70 mL/min.

Mortality was not significantly different between low-flux and high-flux dialyzers in the HEMO study, which was a large randomized controlled study (9). Increases in the dialysis dose and clearance of small-molecular-weight substances were not associated with improved outcomes in the hemodialysis patients in the HEMO study. However, the superiority of high-flux dialyzers was found through subgroup analysis. In patients with a longer dialysis duration, more than 3.7 years, high-flux dialyzers were associated with significantly better survival than low-flux dialyzers (24). In addition, after adjustment for residual kidney function and duration of dialysis, the pre-hemodialysis β2MG level was found to be an independent predictor of mortality (25). In Japan, kidney transplantation is performed in selected patients. In 2010, only 1,485 kidney transplantations were performed, representing 0.5% of all Japanese dialysis patients (26). Therefore, the median dialysis duration of the participants in the present study was 6 years. Another large randomized controlled study, the Membrane Permeability Outcome (MPO) study, showed that high-flux dialyzers were associated with significantly better survival than low-flux dialyzers in patients with diabetes or serum albumin levels <4.0 g/dL (27). Furthermore, cardiovascular mortality, which is a major cause of death in dialysis patients, was found to be reduced in patients treated with high-flux dialyzers in a meta-analysis (28). A systematic review also found significant benefits of high-flux dialyzers on all-cause mortality for certain pre-specified conditions, such as a serum albumin level <4 g/dL, a maintenance hemodialysis duration >3.7 years, and presence of diabetes or arteriovenous fistula (29). Based on these results, the updated 2015 Kidney Disease Outcomes Quality Initiative guidelines recommend the use of biocompatible high-flux hemodialysis membranes for hemodialysis (22). However, in the present study, more than 98% of the patients were treated with high-flux or protein-leaking dialyzers and the patients had a mean serum albumin level of 3.7 g/dL and a longer duration of dialysis. High-flux dialyzers might be beneficial in the present population. Therefore, the current study compared the effects of high-flux and protein-leaking dialyzers on patient outcome.

Recently, not only middle-molecular-weight toxins, such as β2MG (molecular weight, 11.8 kDa), but also high-molecular-weight toxins, such as α1-microglobulin (molecular weight, 33.0 kDa) and protein-bound uremic toxins, have been targeted for removal in hemodialysis patients, which might improve prognosis (30, 31). The removal of middle-sized toxins depends on both dialyzer permeability and treatment modality. Therefore, online hemodiafiltration using high-flux dialyzers is a more efficient treatment modality than low-flux and high-flux hemodialysis. In particular, high-volume post-dilution online hemodiafiltration, defined as a convective volume of at least 23 L/session, could permit greater removal of both uremic toxins and improve outcomes (32, 33). It offers the best clearance of small- and middle-sized molecules and is widely used in Japan and some European countries. Unfortunately, however, online hemodiafiltration cannot be the treatment of choice for all maintenance hemodialysis patients, and it tends not to be widely available in many countries. With the limitations of high-volume post-dilution online hemodiafiltration, hemodialysis with a novel type of dialyzer that has a larger pore size than standard high-flux dialyzers might allow better removal of protein-bound and middle-molecular-weight toxins (34). Protein-leaking dialyzers are characterized by not only a higher β2MG clearance rate, but also a higher ultrafiltration coefficient (i.e., 40–60 mL/h/mmHg/m^2^) and a sieving coefficient of albumin < 0.03 (35). Furthermore, another novel class of membranes is called “medium cut-off (MCO) membrane dialyzers” or “super-flux” membrane dialyzers” and they have recently been designed and incorporated into clinical practice during hemodialysis treatments (36, 37).

However, protein-leaking dialyzers have been used in Japanese hemodialysis patients since 2005. In 2005, the concept of the “high-performance membrane” (HPM) dialyzer, which was unique to Japan, was established. HPM dialyzers are defined as having high hydraulic permeability, high solute permeability, particularly for middle-molecular-weight molecules and uremic toxins with molecular weights of 10–30 kDa, high biocompatibility, and β2MG clearance >50 mL/min (13). HPMs have larger pores than high-flux membranes, which means that they can remove small, medium, and large molecules, including low-molecular-weight proteins and small amounts of albumin (38). The optimal pore size should prevent the loss of >3 g of albumin per session with the standard hemodialysis procedure in Japan of a blood flow rate of 200 mL/min and dialysate flow rate of 500 mL/min (13, 38). In the present study, more than 90% of the hemodialysis patients were treated with HPM dialyzers, in accordance with JSDT recommendations on HPM dialyzer use (13). Therefore, HPM dialyzers and protein-leaking membrane dialyzers belong to the same class of dialyzer, and these membranes can be used only in the hemodialysis modality. Protein-leaking dialyzers are reported to be non-inferior to high-volume post-dilution online hemodiafiltration for removing protein-bound and middle-molecule-weight toxins (39–41), and they could therefore be an option for long-term hemodialysis patients. However, those previous studies were short-term, compared solute clearance, and did not investigate outcomes. The present study revealed the superiority of protein-leaking dialyzers over high-flux dialyzers. Furthermore, we determined that type V dialyzers are superior to type IV dialyzers, even though they are in the same “protein-leaking” category. In addition, the findings should be broadly generalizable to the Japanese dialysis population and may be helpful in other countries where low-flux membrane dialyzers are used.

Six types of dialyzer membrane materials were used in the present study: cellulose triacetate (CTA), ethylene-vinyl alcohol co-polymer (EVOH), polyester polymer alloy (PEPA), polyethersulfone (PES), polymethylmethacrylate (PMMA), and polysulfone (PS) (10, 12). EVOH, PEPA, and PMMA are not frequently used in other countries, whereas CTA, PES and PS dialyzers are frequently used worldwide. In the present study, over half of the patients (57.9%) underwent hemodialysis with a PS membrane, followed by PES (15.3%), CTA (14.5%), PEPA (7.5%), PMMA (3.9%), and EVOH (0.9%). Although many types of dialyzers have been used in Japan, it is similar to other countries in that PS membranes are the most frequently used. However, the use of type V dialyzers increased from 21.3% in 2010 to 35.3% in 2017 (42). Therefore, further investigation is needed to clarify whether the increasing number of patients treated with type V dialyzers has improved prognosis.

There are several limitations to this study. First, selection bias might have occurred. The numbers of patients differed among the five dialyzer groups in the Japanese classification due to the collection of data via annual surveys and the observational study design. The patients in the low-flux and type I dialyzer groups had poor nutritional status and a higher rate of comorbid CVD. Furthermore, mortality rates might have varied among the participating facilities due to differences in practice and patient populations. The number of patients who were waiting for kidney transplantation, which might represent general health status, could not be collected. However, we confirmed the superiority of the protein-leaking dialyzer or type V dialyzer after propensity score matching analysis. Second, the duration of this study was 3 years, which was relatively short. Therefore, a further prospective randomized controlled trial with a longer duration is needed to clarify the superiority of type V and protein-leaking dialyzers. Third, unknown or unmeasured confounders may have affected the association between dialyzer type and mortality. We did not obtain data on residual kidney function, which could be a possible confounder. Finally, we excluded patients treated with hemodiafiltration to eliminate a modality bias and account for the small number of such patients in 2010 in Japan (18). However, hemodiafiltration is considered more efficient at using high-flux dialyzers, and the number of patients treated with hemodiafiltration is growing in Japan. Therefore, further prospective studies comparing protein-leaking hemodialysis vs. high-volume hemodiafiltration are needed to evaluate differences across treatment modalities.

## Conclusions

In conclusion, dialyzer type, classified by β2MG clearance, was significantly associated with 3-year mortality in this large national cohort study of Japanese dialysis patients. Based on our findings, protein-leaking dialyzers might be beneficial in hemodialysis patients. Although type IV and V dialyzers are both classified as protein-leaking membrane dialyzers, this study indicated the superiority of type V dialyzers. Randomized controlled studies are warranted to determine whether the higher β2MG clearance of protein-leaking membrane dialyzers improves outcomes for hemodialysis patients.

## Data Availability Statement

Publicly available datasets were analyzed in this study. This data can be found here: Japanese Society for Dialysis Therapy https://www.jsdt.or.jp/.

## Author Contributions

MA, SN, and IM: conceived and designed the experiments. MA: performed the experiments and wrote the paper. AW and MA: analyzed the data. MA and IM: contributed reagents, materials, and analysis tools. KN and HN: supervision. All authors contributed to the article and approved the submitted version.

## Conflict of Interest

The authors declare that the research was conducted in the absence of any commercial or financial relationships that could be construed as a potential conflict of interest.

## Publisher's Note

All claims expressed in this article are solely those of the authors and do not necessarily represent those of their affiliated organizations, or those of the publisher, the editors and the reviewers. Any product that may be evaluated in this article, or claim that may be made by its manufacturer, is not guaranteed or endorsed by the publisher.
