# Supplementary material for: Dialyzer Classification and Mortality in Hemodialysis Patients: A 3-Year Nationwide Cohort Study
Source: Front Med (Lausanne). 2021 Aug 27;8:740461. doi: 10.3389/fmed.2021.740461 (PMC8429602; doi:10.3389/fmed.2021.740461)
Supplement: Supplementary file 1 [file Table_1.docx]

Supplementary Material

# Supplementary Table 1. Dialyzer classification

| International classification | Japanese classification | β2MG clearance (mL/min) | UN clearance (mL/min) | UFR (mL/h/mmHg) |
| --- | --- | --- | --- | --- |
| Low flux | I | < 10 | ≥ 125 | < 15 |
| High flux | II | ≥ 10-30 | ≥ 150 | ≥ 15 |
| High flux | III | ≥ 30-50 | ≥ 150 | ≥ 15 |
| Protein-leaking | IV | ≥ 50-70 | ≥ 150 | ≥ 50 |
| Protein-leaking | V | ≥ 70 | ≥ 150 | ≥ 50 |

β2MG, β_2_-microglobulin; UFR, ultrafiltration rate; UN, urea nitrogen.

# Supplementary Table 2. Names and characteristics of the dialyzers used in the present study

| Material | Company | Japanese model | Surface area (m^2^) | UFR ^a^ (mL/mmHg/h) | Clearance (mL/min) ^a,b^ | | |  | Sieving coefficient | | Japanese classification |
| --- | --- | --- | --- | --- | --- | --- | --- | --- | --- | --- | --- |
|  |  |  |  |  | UN | Cr | β2MG |  | β2MG | Albumin |  |
| CTA | Nipro Corp. | FB-EG | 1.5 | 12.8 | 191 | 180 | 8 |  | 0.33 | NA | I |
|  |  | FB-E, FB-G | 1.5 | 31.2 | 197 | 192 | 20 |  | 0.62 | NA | II |
|  |  | FB-Pβ | 1.5 | 58.4 | 198 | 192 | 53 |  | 0.80 | NA | III |
|  |  | FB-Uβ | 1.5 | 60.1 | 198 | 192 | 65 |  | 0.99 | 0.01 | IV |
|  |  |  |  |  |  |  |  |  |  |  |  |
| EVOH | Kawasumi Laboratories, Inc. | KF | 1.5 | 8.7 | 179 | 157 | 17.5 |  | NA | 0.02 | I |
|  |  | kf-m | 1.5 | 11.5 | 175 | 155 | 16.6 |  | 0.67 | 0.03 | II |
|  |  | EK | 1.6 | 37.7 | 185 | 176 | 44.6 |  | NA | <0.01 | III |
|  |  |  |  |  |  |  |  |  |  |  |  |
| PEPA | Nikkiso Co., Ltd. | FLX, FDY | 1.5 | 50.0 | 190 | 183 | 65 |  | NA | NA | IV |
|  |  | FDY-GW | 2.1 | 63.0 | 194 | 189 | 71 |  | NA | NA | V |
|  |  |  |  |  |  |  |  |  |  |  |  |
| PES | JMS Co. | BP-N | 1.5 | 43.0 | 190 | 178 | 75 |  | NA | 0.01 | V |
|  | Nipro Corp. | PES-DS | 1.5 | 34.7 | 190 | 181 | 48 |  | 0.98 | 0.01 | III |
|  |  | PES-Eα | 1.5 | 57.9 | 194 | 192 | 65 |  | 0.99 | <0.01 | IV |
|  |  | PES-Dα | 1.5 | 86.9 | 199 | 198 | 77 |  | 0.99 | <0.01 | V |
|  |  |  |  |  |  |  |  |  |  |  |  |
| PMMA | Toray Industries, Inc. | BK-U | 1.6 | 31.0 | 187 | 169 | 51 |  | NA | 0.03 | IV |
|  |  | BG-U, BG-PQ | 1.6 | 31.0 | 190 | 172 | 55 |  | NA | 0.009 | IV |
|  |  |  |  |  |  |  |  |  |  |  |  |
| PS | Fresenius Medical Care Japan KK | F-HPS | 1.4 | 49.0 | 197.5 | 182.1 | 48.7 |  | 0.8 | NA | IV |
|  |  | FX | 1.4 | 50.0 | 195.6 | 182.4 | 68.1 |  | 0.945 | NA | V |
|  | Kawasumi Laboratories, Inc. | PS-H | 1.6 | 68.0 | 187 | 177 | 62.6 |  | NA | NA | IV |
|  |  | PS-UW | 1.6 | 66.0 | 192 | 179 | 56.2 |  | 0.83 | <0.01 | IV |
|  |  | PS-MW | 1.6 | 58.0 | 191 | 178 | 48.4 |  | NA | <0.01 | III |
|  | Asahi Kasei-Kuraray Medical Co. Ltd. | APS-UA | 1.5 | 42.0 | 196 | 191 | 48 |  | 0.7 | <0.002 | III |
|  |  | APS-SA | 1.5 | 63.0 | 196 | 191 | 68 |  | 0.85 | 0.002 | IV |
|  |  | APS-E | 1.5 | 66.0 | 190 | 181 | 74 |  | 0.87 | 0.008 | V |
|  |  | VPS | 1.5 | 63.0 | 196 | 190 | 62 |  | 0.82 | 0.002 | IV |
|  | Toray Industries, Inc. | TS | 1.6 | 54.0 | 194 | 183 | 59 |  | NA | 0.006 | IV |
|  |  | TS-PX | 1.6 | 50.0 | 196 | 187 | 76 |  | NA | 0.011 | V |

All data were provided by the manufacturer. β2MG, β_2_-microglobulin; Cr, creatinine; CTA, cellulose triacetate; EVOH, ethylene-vinyl alcohol co-polymer; PEPA, polyester polymer alloy; PES, polyethersulfone; PMMA, polymethylmethacrylate; PS, polysulfone; UFR, ultrafiltration rate; UN, urea nitrogen. a Plasma was used as pseudoblood in the experiment. b Clearances were measured under the following conditions: blood flow rate = 200 mL/min, dialysis fluid flow rate = 500 mL/min, and ultrafiltration flow rate per unit surface area =10 mL/min/m^2^.

# Supplementary Table 3. Proportion of each categorical variable in maintenance hemodialysis patients

| Variables | Proportion (%) |
| --- | --- |
| Dialysis duration (years) |  |
| < 2 | 22.6 |
| ≥ 2-5 | 24.9 |
| ≥ 5-10 | 25.9 |
| ≥ 10-15 | 12.9 |
| ≥ 15-20 | 6.5 |
| ≥ 20-25 | 3.5 |
| ≥ 25 | 3.7 |
| Body mass index (kg/m^2^) |  |
| < 16 | 4.1 |
| ≥ 16-18 | 12.2 |
| ≥ 18-20 | 22.2 |
| ≥ 20-22 | 23.8 |
| ≥ 22-24 | 17.9 |
| ≥ 24-26 | 10.2 |
| ≥ 26-28 | 5.0 |
| ≥ 28 | 4.6 |
| Serum albumin (g/dL) |  |
| < 3.0 | 4.2 |
| ≥ 3.0-3.5 | 21.6 |
| ≥ 3.5-4.0 | 50.9 |
| ≥ 4.0-4.5 | 21.9 |
| ≥ 4.5 | 1.4 |
| Kt/V |  |
| < 0.8 | 1.0 |
| ≥ 0.8-1.0 | 3.5 |
| ≥ 1.0-1.2 | 11.8 |
| ≥ 1.2-1.4 | 32.9 |
| ≥ 1.4-1.6 | 26.1 |
| ≥ 1.6-1.8 | 14.7 |
| ≥ 1.8-2.0 | 6.4 |
| ≥ 2.0 | 3.6 |
| nPCR (g/kg/day) |  |
| < 0.6 | 6.1 |
| ≥ 0.6-0.8 | 29.5 |
| ≥ 0.8-1.0 | 41.7 |
| ≥ 1.0-1.2 | 18.7 |
| ≥ 1.2 | 4.0 |
| %CGR (%) |  |
| < 60 | 13.7 |
| ≥ 60-80 | 16.7 |
| ≥ 80-100 | 25.6 |
| ≥ 100-120 | 26.8 |
| ≥ 120-140 | 13.9 |
| ≥ 140 | 3.3 |

%CGR, percent creatinine generation rate; nPCR, normalized protein catabolic rate.

**Supplementary Table 4.** Hazard ratios and 95% confidence intervals for variables evaluated as potential predictors of mortality among all patients

|  | HR | 95%CI | P value |
| --- | --- | --- | --- |
| Sex |  |  |  |
| Male | 1.000 | Reference | - |
| Female | 0.919 | 0.892-0.964 | < 0.0001 |
| Age (years) |  |  |  |
| 1-year increase | 1.064 | 1.063-1.065 | < 0.0001 |
| Dialysis duration (years) |  |  |  |
| < 2 | 0.935 | 0.913-0.959 | < 0.0001 |
| ≥ 2-5 | 1.000 | Reference | - |
| ≥ 5-10 | 1.031 | 1.008-1.055 | 0.008 |
| ≥ 10-15 | 0.909 | 0.883-0.936 | < 0.0001 |
| ≥ 15-20 | 0.761 | 0.731-0.792 | < 0.0001 |
| ≥ 20-25 | 0.713 | 0.676-0.752 | < 0.0001 |
| ≥ 25 | 0.901 | 0.859-0.945 | < 0.0001 |
| Primary kidney disease |  |  |  |
| Glomerulonephritis | 1.000 | Reference | - |
| Diabetic nephropathy | 1.501 | 1.471-1.531 | < 0.0001 |
| Nephrosclerosis | 1.564 | 1.517-1.613 | < 0.0001 |
| Polycystic kidney disease | 0.860 | 0.813-0.910 | < 0.0001 |
| Others | 1.285 | 1.250-1.321 | < 0.0001 |
| Presence or absence of DM |  |  |  |
| Absence of DM | 1.000 | Reference | - |
| Presence of DM | 1.271 | 1.249-1.292 | < 0.0001 |
| Comorbid CVD |  |  |  |
| No comorbid CVD | 1.000 | Reference | - |
| Comorbid CVD | 2.086 | 2.049-2.123 | < 0.0001 |
| Kt/V |  |  |  |
| < 0.8 | 3.199 | 2.999-3.414 | < 0.0001 |
| ≥ 0.8-1.0 | 2.018 | 1.937-2.102 | < 0.0001 |
| ≥ 1.0-1.2 | 1.425 | 1.386-1.465 | < 0.0001 |
| ≥ 1.2-1.4 | 1.000 | Reference | - |
| ≥ 1.4-1.6 | 0.989 | 0.966-1.013 | 0.384 |
| ≥ 1.6-1.8 | 0.948 | 0.922-0.976 | 0.0003 |
| ≥ 1.8-2.0 | 0.849 | 0.814-0.884 | < 0.0001 |
| ≥ 2.0 | 0.771 | 0.730-0.815 | < 0.0001 |
| β_2_-microglobulin (mg/L) |  |  |  |
| 1 mg/L increase | 1.021 | 1.019-1.023 | < 0.0001 |
| C-reactive protein (mg/dL) |  |  |  |
| 1 mg/dL increase | 1.104 | 1.010-1.11 | < 0.0001 |
| Hemoglobin (g/dL) |  |  |  |
| 1 g/dL increase | 0.822 | 0.817-0.828 | < 0.0001 |
| Body mass index (kg/m^2^) |  |  |  |
| < 16 | 2.889 | 2.785-2.996 | < 0.0001 |
| ≥ 16-18 | 1.766 | 1.716-1.818 | < 0.0001 |
| ≥ 18-20 | 1.264 | 1.231-1.298 | < 0.0001 |
| ≥ 20-22 | 1.000 | Reference | - |
| ≥ 22-24 | 0.842 | 0.816-0.868 | < 0.0001 |
| ≥ 24-26 | 0.732 | 0.704-0.762 | < 0.0001 |
| ≥ 26-28 | 0.669 | 0.634-0.706 | < 0.0001 |
| ≥ 28 | 0.614 | 0.580-0.651 | < 0.0001 |
| Serum albumin (g/dL) |  |  |  |
| < 3.0 | 4.651 | 4.140-5.224 | < 0.0001 |
| ≥ 3.0-3.5 | 2.158 | 2.115-2.203 | < 0.0001 |
| ≥ 3.5-4.0 | 1.000 | Reference | - |
| ≥ 4.0-4.5 | 0.561 | 0.545-0.578 | < 0.0001 |
| ≥ 4.5 | 0.463 | 0.412-0.520 | < 0.0001 |
| nPCR (g/kg/day) |  |  |  |
| < 0.6 | 2.478 | 2.404-2.555 | < 0.0001 |
| ≥ 0.6-0.8 | 1.433 | 1.404-1.463 | < 0.0001 |
| ≥ 0.8-1.0 | 1.000 | Reference | - |
| ≥ 1.0-1.2 | 0.823 | 0.800-0.846 | < 0.0001 |
| ≥ 1.2 | 0.868 | 0.824-0.914 | < 0.0001 |
| %CGR (%) |  |  |  |
| < 60 | 1.983 | 1.934-2.033 | < 0.0001 |
| ≥ 60-80 | 1.489 | 1.385-1.601 | < 0.0001 |
| ≥ 80-100 | 1.403 | 1.368-1.439 | < 0.0001 |
| ≥ 100-120 | 1.000 |  |  |
| ≥ 120-140 | 0.697 | 0.670-0.725 | < 0.0001 |
| ≥ 140 | 0.671 | 0.624-0.721 | < 0.0001 |

%CGR, percent creatinine generation rate; CVD, cardiovascular disease; DM, diabetes mellitus; nPCR, normalized protein catabolic rate.

**Supplementary Table 5.** Hazard ratios (95% CIs) for all-cause mortality among the 3 types of dialyzers in 238,321 maintenance hemodialysis patients, determined using standard Cox proportional hazards regression.

| Dialyzer type | Unadjusted | | |  | Adjusted for basic factors ^a^ | | |  | Adjusted for basic factors and dialysis dose ^b^ | | |  | Adjusted for basic factors, dialysis dose, and nutrition- and inflammation-related factors ^c^ | | |
| --- | --- | --- | --- | --- | --- | --- | --- | --- | --- | --- | --- | --- | --- | --- | --- |
|  | HR | 95% CI | P value |  | HR | 95% CI | P value |  | HR | 95% CI | P value |  | HR | 95% CI | P value |
| Low flux | 1.88 | 1.76-2.00 | < 0.0001 |  | 1.47 | 1.37-1.57 | < 0.0001 |  | 1.20 | 1.31-1.50 | < 0.0001 |  | 1.12 | 1.03-1.22 | 0.009 |
| High flux | 1.00 | Reference | - |  | 1.00 | Reference | - |  | 1.00 | Reference | - |  | 1.00 | Reference | - |
| Protein-leaking | 0.78 | 0.74-0.82 | < 0.0001 |  | 0.83 | 0.81-0.85 | < 0.0001 |  | 0.89 | 0.86-0.91 | < 0.0001 |  | 0.95 | 0.92-0.98 | 0.006 |

a, Adjusted for age, sex, dialysis vintage, presence or absence of diabetes, and cardiovascular disease; b, adjusted for basic factors, Kt/V, and β_2_-microglobulin; c, adjusted for basic factors, dialysis dose, hemoglobin, serum albumin, β_2_-microglobulin, C-reactive protein, calcium, phosphate, intact parathyroid hormone, normalized protein catabolic rate, body mass index, and percent creatinine generation rate. CI, confidence interval; HR, hazard ratio.

**Supplementary Table 6.** Hazard ratios (95% CIs) for all-cause mortality among the 5 types of dialyzers in 238,321 maintenance hemodialysis patients, determined using standard Cox proportional hazards regression.

| Dialyzer type | Unadjusted | | |  | Adjusted for basic factors ^a^ | | |  | Adjusted for basic factors and dialysis dose ^b^ | | |  | Adjusted for basic factors, dialysis dose, and nutrition- and inflammation-related factors ^c^ | | |
| --- | --- | --- | --- | --- | --- | --- | --- | --- | --- | --- | --- | --- | --- | --- | --- |
|  | HR | 95% CI | P value |  | HR | 95% CI | P value |  | HR | 95% CI | P value |  | HR | 95% CI | P value |
| I | 2.35 | 2.21-2.49 | < 0.0001 |  | 1.65 | 1.55-1.75 | < 0.0001 |  | 1.41 | 1.31-1.50 | < 0.0001 |  | 1.10 | 1.02-1.19 | 0.015 |
| II | 2.09 | 1.95-2.21 | < 0.0001 |  | 1.52 | 1.42-1.62 | < 0.0001 |  | 1.37 | 1.27-1.47 | < 0.0001 |  | 1.10 | 1.02-1.39 | 0.014 |
| III | 1.17 | 1.11-1.23 | < 0.0001 |  | 1.07 | 1.02-1.12 | 0.0008 |  | 1.03 | 0.97-1.09 | 0.230 |  | 0.96 | 0.91-1.01 | 0.063 |
| IV | 1.00 | Reference | - |  | 1.00 | Reference | - |  | 1.00 | Reference | - |  | 1.00 | Reference | - |
| V | 0.64 | 0.63-0.65 | < 0.0001 |  | 0.83 | 0.81-0.85 | < 0.0001 |  | 0.86 | 0.83-0.88 | < 0.0001 |  | 0.91 | 0.88-0.94 | < 0.0001 |

a, Adjusted for age, sex, dialysis vintage, presence or absence of diabetes, and cardiovascular disease; b, adjusted for basic factors, Kt/V, and β_2_-microglobulin; c, adjusted for basic factors, dialysis dose, hemoglobin, serum albumin, β_2_-microglobulin, C-reactive protein, calcium, phosphate, intact parathyroid hormone, normalized protein catabolic rate, body mass index, and percent creatinine generation rate. CI, confidence interval; HR, hazard ratio.
